# Spelling and Meaning of Compounds in the Early School Years through Classroom Games: An Intervention Study

**DOI:** 10.3389/fpsyg.2017.02071

**Published:** 2017-11-29

**Authors:** Styliani N. Tsesmeli

**Affiliations:** Division of Psychology, Department of Primary Education, University of Patras, Rion, Greece

**Keywords:** intervention, spelling, morphology, compounds, classroom games

## Abstract

The study aimed to evaluate the intervention effects on spelling and meaning of compounds by Greek students via group board games in classroom settings. The sample consisted of 60 pupils, who were attending the first and second grade of two primary schools in Greece. Each grade-class was divided into an intervention (*N* = 29 children) and a control group (*N* = 31 children). Before intervention, groups were evaluated by standardized tests of reading words/pseudowords, spelling words, and vocabulary. Students were also assessed on compound knowledge by a word analogy task, a meaning task and a spelling task. The experimental design of the intervention included a pre-test, a training program, and a post-test. The pre- and post-assessments consisted of the spelling and the meaning tasks entailing equally morphologically transparent and opaque compounds. The training program was based on word families (*N* = 10 word families, 56 trained items, 5 sessions) and aimed to offer instruction of morphological decomposition and meaning of words. The findings showed that training was effective in enhancing the spelling and most notably the meaning of compounds. A closer inspection of intervention data in terms of morphological transparency, revealed that training group of first graders improved significantly both on transparent and opaque compounds, while the degree of gains was larger on opaque items for the second graders. These findings are consistent with the experimental literature and particularly optimistic for the literacy enhancement of typically developing children in regular classrooms.

## Introduction

Over the last decades, morphology has been receiving increasing attention among studies concerning children’s literacy acquisition (Carlisle, 2003; Nunes and Bryant, 2006; Sénéchal and Kearnan, 2007; Berninger et al., 2010; Bowers et al., 2010; Kirby et al., 2012; Nagy et al., 2014). Various aspects of morphological processing has started to be an important aspect of linguistic enquiry. Morphological awareness has been acknowledged as an essential skill in language development for both typical and non-typical readers, while morphological instruction is recognized to be beneficial for the enhancement of reading, spelling, vocabulary, and reading comprehension of students, especially those with literacy difficulties (Deacon et al., 2006; Bowers et al., 2010; Goodwin and Ahn, 2010, 2013; Gilbert et al., 2013; McCutchen et al., 2014).

Compounding is one of the richest sources of word formation in everyday language and scientific terminology (Ralli, 2005; Scalise and Vogel, 2010). Longitudinal studies have demonstrated that children with typical development acquire simple compounds very early and in a fairly consistent developmental sequence (Nicoladis, 2006). In fact, children primarily begin to treat compound words as single units, and then gradually to understand that a compound may consist of two parts with a meaningful relation between them. However, as soon as literacy is well established and children are progressing to later stages of development, they start out to enrich their language via the compounding process (Berman, 2009).

Beyond these aspects, another significant factor that is thought to influence literacy performance is the level of morphological transparency of compounds. In particular, morphologically transparent words are considered those whose morphemic constituents remain intact during the transformation process (e.g., *blackboard*), thus they are usually visible to an inexperienced reader. Conversely, non-transparent or opaque words entail constituents which undergo a variety of phonological, orthographic or morpho-phonological changes during the word-formation process (e.g., *comprehend, agriculture*), resulting in an obscured internal structure that is not easily discernable by a novice reader. Research findings indicate that transparency affects literacy skills considerably, especially of those who are young children or students with literacy difficulties. In particular, transparent items evoke higher scores than opaque items in spelling and meaning of words, including morphologically complex items (Kuo and Anderson, 2006; Goodwin and Ahn, 2013; Goodwin et al., 2014).

Moreover, semantic transparency of compounds, where the meaning could not be conveyed directly by the two separate lexical bases (e.g., *roadside* vs. *butterfly*, see Brooks and Cid de Garcia, 2015) also affects the individual’s word recognition. In addition, semantic transparency appears to be influenced by ambiguity in morpheme boundaries of a compound and modulated by word length. Recently, Lemhöfer et al. (2011) demonstrated that adult native speakers of Dutch showed a parsing sensitivity when reading long compounds containing a parsing cue, that is a bigram of consonants at the constituent boundary that would be illegal within a morpheme (e.g., *fiet****sb****el* [bicycle bell]), and not in the presence of a legal but ambiguous bigram (e.g., *fiet****sp****omp* [bicycle pump] can be read either as ^∗^*fie****t***-***s****pomp*). On the contrary, bilingual adults of German-Dutch showed this sensitivity on both short and long compounds, a fact that was taken as an indication of a broader use of sub-lexical strategies in compound processing than that of native speakers (p. 365). Similarly, in a relevant study by Bertram et al. (2004) (also in Hyönä et al., 2012, p. 92), the same effect of the ambiguity on legal consonants within a morpheme boundary was found (i.e., when the consonant can either be the last letter of the first constituent or the first letter of the second constituent), but not in the unambiguous condition, where the initial consonant of the second constituent, cannot be the last one of the first constituent. It was also shown that gaze duration was longer when the two vowels at the constituent boundary of long compounds were of the same quality (e.g., *ryöst****öy****ritys* [robbery attempt]) rather than when they were of different quality (e.g., *selk****äo****ngelma* [back problem]), a fact which was regarded as an indication of parsing effects on long compounds (pp. 88–89).

However, these findings do not seem directly relevant for the Greek language, since morpheme boundaries of Greek compounds are rarely ambiguous, given that consonant bigrams that may follow the linking vowel /o/ should be phonologically legal. This process might be better understood by the fact that the most usual type of structure of Greek compounds are mainly stem-based, requiring the first constituent “*to be superficially bare, that is, an item without any suffixal material*” (Ralli, 2013, p. 134), which is mostly followed by the linking vowel /o/. This is true either for morphologically transparent or opaque items ζωοτροφή [*zootrofi*/animal-food], αυγ*ό*τσουφλο [*aνϫotsuflo*-eggshell], see Appendix II in Supplementary Material), while the majority of compounds are typically long (more than eight letters) (see, Appendix II in Supplementary Material), since the majority of the constituents are at least of two syllables. Thus, although, Greek data is sparse on these aspects (Kehayia et al., 1999), it can be hypothesized that parsing may facilitate recognition of compounds, since segmentation cues are already available to readers. In any case, further research is needed on these important aspects of compound processing in Greek.

The focal point of this study is the investigation of compounds during the early school years. Although, Greek is acknowledged to be a regular orthography in terms of reading (Seymour et al., 2003), in terms of spelling is less transparent, since there are a few occasions with one-to-many phoneme–grapheme mappings, which are mainly rule governed. Since these spelling patterns are made comprehensible via the etymological and grammatical word features, spelling can be supported by a progressive acquisition of the rules based on morphology and lexical information (Porpodas, 2006). Moreover, Greek is a language with a rich morphology with lots of polysyllabic and morphologically complex words, while compounding is one of the central ways of word-formation (Ralli, 2005). More than 60,000 compounds are present in the language, usually forming multi-member word-families (Babiniotis, 2016). Compound acquisition, therefore, seems to be of major significance for the spelling and vocabulary development in Greek, even from the early school years, where children encounter an increased number of compounds in their school books (for instance, about 500 compounds are encountered in Grade 1 school books, and 800 compounds in Grade 2 school books).

Greek compounds are normally formed by placing a linking vowel (-o-) between the first and second constituent and are mostly right-headed. In terms of word structure, they are allocated into four main categories (Ralli, 2005, 2013): (i) Stem–Stem, where two lexical stems, along with a linking vowel (-o-) between them and an inflection in the end, form a compound (e.g., χρυσ+*ό*+ψαρ+ο /*chris+o+psar+o/ gold fish*), (ii) Stem–Word, where a lexical stem and a word with the linking vowel (-o-) between them, form the compound (e.g., μελαν+ο+δοχείο/ *melan+o*+ð*ocheio/ inkpot*), (iii) Word–Stem, where a word and a lexical stem with the compound’s inflection are united together to form a compound (e.g., κατω+σέντον+ο/ *kato+senton+o/ undersheet*), and finally, (iv) Word–Word, where two words are united together without a linking vowel between them (e.g., *ξ*ανα+δουλεύω/ *xana*+ð*oulevo/ work again*). According to Ralli (2005, 2013), the majority of compounds in Greek fall in the first two categories, usually forming large word-families, while the other categories are minor and usually non-productive, especially the last one. Since both of the first categories are non-transparent compounds, it can be concluded that the majority of compounds in Greek are morphologically opaque compounds, where the main constituents of the compound are not easily visible to a novice reader, while the last category entailed compounds that are morphologically transparent, thus easily analyzed even by a novice native reader. Greek data in terms of transparency showed that children are also affected by transparency effects, causing noteworthy difficulties to those with impoverished spelling abilities (Rousoulioti, 2011). Given the effects of morphological knowledge to spelling, current research suggests that systematic and sequential instruction of morphology is necessary in the elementary school, even from the earliest years of schooling (Sénéchal and Kearnan, 2007; Reed, 2008).

According to recent theoretical accounts of literacy acquisition (Seymour and Duncan, 2001; Seymour et al., 2003; Duncan et al., 2013) acquisition of morphology in regular orthographies, such as Greek, typically occur earlier than in deep orthographies, such as English, after the grasp of grapheme–phoneme correspondences and the syllabic system of the language. Relevant evidence from other rather regular orthographies, such as French, suggests also an early development of morphological awareness in comparison with English (Casalis and Colé, 2009). According to Seymour and Duncan (2001, p. 296), Greek children can progress rapidly through the foundation and alphabetic phase and approach the morphographic phase, in which morphological structure of words is emphasized, with an inventory of well-defined syllabic units in place, whereas English children require up to 2 years of instruction in order to progress through the above phases. This is empirically true since Greek children typically are able to read and write by the end of their first year of schooling, while acquisition of the morphology is accelerated via the systematic instruction in Greek schools, which involves at least instruction on the inflectional system of the language. Thus, morphological training appears to be important even in the initial phase of literacy acquisition, since young children have to face a large amount of complex words, and learn from very early the complexities of the rich morphological system in Greek. Certainly, this varies in different orthographic systems depending on the nature and characteristics of the morphological system of each alphabetic language, beyond the differences in terms of their phonological system.

On the other hand, it could be argued that children as young as first graders may be too young to grasp the morphological principles. However, relevant data (Casalis and Colé, 2009; Lyster et al., 2016) shows that children are able to use these principles, providing that they are taught in a comprehensible way via multi-sensory methods and not by abstract rules. Besides, data from children with impoverished phonological skills appears to be benefitted from morphological instruction, even in their first grades. For instance, Casalis and Colé (2009) found that morphological awareness training may increase preschoolers’ sensitivity to sounds, a finding which is also reported earlier on older children with phonological difficulties, such as dyslexic teenagers (Arnbak and Elbro, 2000; Tsesmeli, 2002). These findings give further support to morphological training studies with children, even at very early grades of schooling.

Intervention studies on morphological awareness carried out on early readers, even preschoolers, illustrate that young learners are responsive to morphological instruction. A recent study by Ramirez et al. (2014) investigated training effects on a sample of 108 kindergartners with different ability levels taken from six classes from schools of low socio-economical status. The intervention focused only on compounds with semantic and morpho-phonological transparency (i.e., *teapot*). Before training, children were assessed by a standardized vocabulary test, and also by another task where they were asked to produce 10 compounds in a story context, e.g., “*We call a house that is built in a tree, a tree house. What should we call a house that is built on a mountain?*” (p. 58). The intervention took place in classrooms within a 3 months period and included 24 sessions of 30 min each, where children were asked mostly orally to analyze compounds into their morphological constituents and to produce new ones from already known morphemes. The material based on 10 illustrated story-books entailed 10–15 compounds or pseudo-compounds per book (e.g., *Dino-Soccer*). Children were asked to analyze them into morphemes and think how to derive the words’ meaning, or to produce new words (e.g., *Dinohouse*) via assorting pictures which illustrated concrete words (e.g., *house*) with already knows morphemes (e.g., *Dino*). Results showed that children significantly improved their morphological skills and vocabulary over a period of 3 months, with the greatest gains made by children with the lowest performance before the intervention. However, students of medium performance also improved and reached the levels of students of high performance. Moreover, the distance between those with low vocabulary skills and those with higher skills diminished, and it is possible that the intervention assisted in their enhanced performance on the vocabulary assessment. Authors concluded that morphological awareness and vocabulary skills were reciprocally related, which was indicated by the fact that morphological awareness made an independent contribution to the development of vocabulary and that vocabulary made an independent contribution to the development of morphological awareness (Ramirez et al., 2014). However, in this study there were no control groups, and no association with factors of emergent literacy skills.

In another recent study by Apel and Diehm (2014), the effectiveness of intervention on the morphological awareness and literacy skills on a group of 75 students who followed kindergarten (*n* = 27), Grade 1 (*n* = 22), Grade 2 (*n* = 26) was investigated, which compared with a control group of 76 students (kindergarteners = 27, Grade 1 = 21, Grade 2 = 28). The intervention included 32 sessions of 25 min for a period of 8 weeks and focused on awareness of affixes and the relations between base words and their inflected or derived forms. Students from experimental groups were taught in small groups at the school library. Intervention included the teaching of 11 inflectional (e.g., -*ing*) and derivational affixes (e.g., *-ness*) through a variety of educational activities. For instance, students should be able to identify the taught morphemes on target words and produce new words from the already taught morphemes. Findings showed that experimental groups across grades produced statistically significant gains in morphological awareness skills but non-significant gains in literacy skills (reading and reading comprehension), while the control groups did not show any increase in any of these skills. In particular, explicit morphological instruction benefited students with weak morphological awareness abilities at the beginning of the study, who reached their levels of their peers of typical morphological awareness abilities after the intervention.

In another study by Wolter and Dilworth (2014), there were investigated morphological training effects on 20 second grade children with spelling deficits allocated into two intervention groups: (i) a group trained in phonological and orthographic awareness activities, and (ii) a group trained in phonological, orthographic and morphological awareness activities. Morphological activities included mainly inflectional and derivational affixes. Findings of this study indicated that the morphological awareness intervention group increased its performance on standardized spelling, spelling of morphological patterns and a measure of reading comprehension, while there were no differences between the groups on spelling of orthographic patterns.

However, morphological facilitation is feasible not only on deep orthographies, as described above, but also to rather phonologically transparent orthographies, such as Norwegian or French. A recent study by Lyster et al. (2016) on Norwegian-speaking children suggest that early training in morphological awareness can have long-term effects on children’s literacy skills. In particular, preschoolers divided into a phonology group (*N* = 106), a morphology group (*N* = 127), and a control group (*N* = 36) which participated in ordinary pre-school activities. Training for the two experimental groups lasted over 17 weeks’ time. The phonology group participated in activities involving syllable and sound blending or matching words based on alliteration and rhyming. The morphology group received training on suffix and prefix identification on derivations as well as recognition of constituents of compounds (e.g., ‘*skoeske*’/ *shoe box*). Activities also entailed segmenting, deleting or changing the order of compound constituents to create new compounds. Findings showed that children who received morpheme training in preschool improved both reading comprehension and word reading at the end of Grade 1 in comparison with their control group. Most notably, the morpheme trained group exhibited better reading comprehension skills 6 years later, at Grade 6, than the control group, despite the fact that the children in this group had lower phonological skills compared with the control group children before intervention. Relevant findings on French pre-school children are coming from a training study by Casalis and Colé (2009). They found that morphological awareness can be successfully trained before formal schooling, although the training at a pre-school age did not produce significant transfer of learning results on first years’ reading skills, possibly because, as authors stated, children were not involved in print exposure activities.

The above studies conducted mainly in English presented the experimental evidence that morphological training could be of an essential advantage to students of typical and non-typical development, even from the very early grades of schooling. While the main corpus of evidence is less extended to other alphabetical systems (Casalis and Colé, 2009; Tsesmeli, 2010; Lyster et al., 2016), apart from English, intervention studies in this field are still lacking, especially in phonologically regular languages, such as Greek.

The present study aims to examine the efficiency of training in the spelling and meaning of compound words by two experimental groups of young students of first and second grade, who are in the early stages of literacy acquisition. The study extends earlier intervention case-studies in Greek by Tsesmeli (2010) and Tsesmeli and Tsirozi (2015), since it would be important to see how training effects from individuals would be transferred to students of typical development in regular school classes (i.e., Nunes and Bryant, 2006). Moreover, an essential educational aim is to assist “*all children achieve their optimal levels*” (Ramirez et al., 2014, p. 54) in terms of literacy, and help children who are at risk of developing such difficulties to start from very early, due to the accumulative nature of these types of deficiencies (Ramirez et al., 2014). Also, research outcomes would be suggested for educational and school policy to evoke the wider transfer of learning effects.

The training was systematic and sequential and implemented via a variety of board games in small groups in the whole classroom, as being the most appropriate way to teach early learners at their first years of schooling (Brock et al., 2009). Main benefits were the multisensory learning via real objects, the promotion of visual coding strategies and the active participation of the students through recreational and learning targets of their content. The advantages of these multi-modal programs are that they can capture the student’s interest, creating strong motivations for learning and knowledge acquisition (Kast et al., 2011). In the last decades, there have developed a variety of intervention programs for early readers aiming to train early literacy abilities, focusing mainly on the phonological processing of words (McCandliss et al., 2003; Brooks et al., 2007; Kast et al., 2011), and to a lesser extent to other linguistic abilities.

### Main Hypotheses of the Study

The main hypotheses for the training study can be formulated as the following: (i) Each experimental group would enhance considerably the spelling performance of compounds after training, as an effect of the intervention (Tsesmeli, 2002; Nunes and Bryant, 2006; Tsesmeli and Seymour, 2009; Wolter and Dilworth, 2014); (ii) Transparent compounds are assumed to be spelled more successfully than opaque items (Carlisle, 1987; Kuo and Anderson, 2006; Goodwin et al., 2014; To et al., 2014) before the intervention, however, it is anticipated that the training would increase performance on compounds by each compound category (Tsesmeli and Seymour, 2009); (iii) Each experimental group would increase substantially the meaning performance of compounds after training, due to intervention (Bowers and Kirby, 2010; Ramirez et al., 2014); (iv) It will be investigated whether experimental groups would recognize the internal structure of the compounds to a greater extent after the intervention than the control groups (Anglin, 1993; Tsesmeli and Koutselaki, 2013); (v) Differences of training effects between spelling and meaning of compounds will also be assessed in order to be identified particular profiles for each experimental group (Tsesmeli and Koutselaki, 2013); (vi) Generalization effects will be finally evaluated based on the hypothesis that untrained pseudo-compounds of similar structure would induce transfer-of-learning effects (Wysocki and Jenkins, 1987; Nunes and Bryant, 2006) after intervention, since they include common stems with the trained items (Bowers and Kirby, 2010).

## Materials and Methods

### Participants

Participants for the study were 60 students (34 males and 26 females) who were attending the first and the second grades of two primary schools in the prefecture of Achaia in Peloponnese, Greece. Students in each grade were already allocated to their regular classrooms situated in two different schools. A possible variation within classrooms in terms of cognitive/academic abilities is usual, since public schools in Greece accept children based mainly on chronological criteria. Since the basic aim of the study was to evaluate the possible efficiency of an intervention program to regular classes within the schools, no other selection criteria were applied to the groups. The choice in relation to which class of each grade would be the experimental group was based on the standardized measures of reading and spelling abilities (see the section The Preliminary Assessment) and teachers’ motivation to participate in the training program. Thus, teachers who had in their classes more cases with lower performance than the mean, appear to be more willing to give their consent to the program. However, there were no students with a *z*-score above 1.50 SD on these skills, apart from two cases equally belonging to the experimental and control groups of Grade 1. These characteristics are presented in terms of groups in more detail^1^ as follows:

(i) Grade 1-Experimental group (*N* = 14): They were students who participated in the training program with a mean chronological age of 6.48 years (sd: 0.47). Children’s variation in terms of reading words (mean: -0.31, range: 1.72 to -1.45, sd: 1.07) and non-words (mean: -0.54, range: 1.21 to -1.60, sd: 0.77) based on *z*-scores on standardized measures of reading ability was within the normal range, while three children were at the lower normal limit (-1.45 SD) on both abilities, and only one child had a *z*-score of -1.60 SD on reading non-words.
(ii) Grade 1-Control group (*N* = 15): They were students who did not take part in any intervention study, apart from the regular classroom teaching, and had a mean chronological age of 6.40 years (sd: 0.46). Children’s variation in terms of reading words (mean: 0.29, range: 1.72 to -1.84, sd: 0.85) and non-words (mean: 0.50, range: 1.56 to -1.07, sd: 0.93) based on *z*-scores on standardized measures of reading ability was within the normal range, while only one child had a *z*-score of -1.84 SD on reading words.
(iii) Grade 2-Experimental group (*N* = 15) entailed students who took part in the intervention and had a mean chronological age of 7.34 years (sd: 0.42). Children’s variation in terms of spelling words (mean: -0.53, range: 0.61 to -1.40, sd: 0.63) and vocabulary acquisition (mean: -0.64, range: 0.60 to -1.40, sd: 0.63) based on *z*-scores on standardized measures of these skills was within the normal range, while three children were at the lower normal limit (-1.40 SD) on both abilities.
(iv) Grade 2-Control group (*N* = 16) consisted of students who did not participate in any intervention study, apart from the regular classroom teaching and had a mean chronological age of 7.32 years (sd: 0.42). Children’s variation in terms of spelling words (mean: 0.50, range: 1.77 to -1.12, sd: 1.03) and vocabulary acquisition (mean: 0.60, range: 1.80 to -0.75, sd: 0.90) based on *z*-scores on standardized measures of these skills was within the normal range.

All students were Greek monolinguals of average socioeconomic background and they had no mental, hearing, visual, or serious health problems. Their participation was secured after parents’ written consent in the study.

### The Preliminary Assessment

All students, before initiation of the intervention, were given a series of standardized psychometric tests so as to evaluate their reading, spelling, and written vocabulary abilities. Grade 1 students were given only the standardized measures of reading ability, since there are no standardized tests in spelling and written vocabulary in Greek for this grade. Also, the standardized test of reading ability was not given to Grade 2 students, due to the possible ceiling effects for this age. These tests are exhibited as follows:

*(a) Reading ability of words/non-words* was evaluated by the Test of Reading Performance (TORP; Padeliadu and Sideridis, 2000) which is an untimed comprehensive test of reading ability in Greek. Each student had to read 40 single words derived from school reading books and structured by word frequency and ascending phonological difficulty. Next, every pupil had to decode 19 pseudowords of ascending phonological difficulty. Both reading tests were instructed individually to Grade 1 students at a quiet room within the school. Scoring was based on reading accuracy in terms of target decoding and stress, without time constraints.
*(b) Spelling skills of words* was estimated by the Test of Spelling Ability by Mouzaki et al. (2007), which consists of 60 single words taken also from school books and ordered in terms of word length and increasing phonographic difficulty. Words were dictated in sentences to students of Grade 2 by their classroom teachers.
*(c) Understanding of written vocabulary* in written speech was assessed by the test of Tafa (1995) where each student had to complete 42 open-ended sentences by choosing the suitable word from a multiple choice scheme that fits in the sentence syntactically and semantically. Items were instructed to students of Grade 2 by their teachers in the classroom.

**Table 1** presents the results of psychometric evaluation for the participants based on mean accuracy rates. The one-way analysis of variance between the experimental and control group of Grade 1 showed that their mean performance on reading single words [*F*(1,28) = 2.885, ns] did not differ with each other, while in terms of reading non-words, the experimental group had significantly lower performance than the control group [*F*(1,28) = 10.896, *p* < 0.01]. Similarly, analysis of variance between the two groups of Grade 2 showed that their mean performance on spelling single words [*F*(1,30) = 11.368, *p* < 0.01] and acquisition of written vocabulary [*F*(1,30) = 19.231, *p* < 0.001] differed significantly in favor of the control group.

**Table 1:** Psychometric data.

	Grade 1 Experimental	Grade 1 Control	Grade 2 Experimental	Grade 2 Control
Reading words	87.14 (6.78)	91.00 (5.41)		
Reading non-words	36.84 (23.17)	68.42 (27.92)		
Spelling words			28.97 (7.30)	40.38 (11.94)
Meaning words			39.95 (11.30)	62.00 (16.10)


*Accuracy means (%) (standard deviations in parentheses).*

### Experimental Assessments

For the purpose of the study, three experimental tasks were developed. Experimental stimuli were chosen from Grades 1 and 2 school books after being selected from a large pool of compounds regarded to represent a wide range of morphological patterns found in school texts. Word analogy and the meaning tasks were delivered individually to students, while the spelling task was dictated to them by their teachers in classrooms. These are described in detail as follows:

#### (1) Word Analogy Task (*N* = 24/40 Items)

The task aimed to evaluate via word analogies the explicit awareness of oral production of compounds. The task entailed 24 items for Grade 1 and 40 items for Grade 2. All items in this task were equally divided in pairs of compounds. Participants had to discriminate compound transformations in the first word-pair and apply the same transformation to the second pair. Items involved morphologically transparent words (*n* = 6), morphologically opaque words (*n* = 6), compound words including bound stems of ancient Greek origin (*n* = 4), and prefixed compounds (*n* = 4). Examples of these items in the above categories are shown in Appendix I in Supplementary Material. The task was delivered orally via lap-top recordings by a native speaker and instructed to every student by the investigator. Every accurate response was given 1 point and every inaccurate was given 0 points.

#### (2) Spelling of Pair of Compounds (*N* = 60 Items)

Spelling of compounds was evaluated by a list of 60 items, equally divided in pairs of compounds. The items were also allocated to three categories in terms of transparency and lexicality of compounds, as follows: (i) pairs of transparent compounds (*n* = 10 pairs), where no change on the morphological constituents was apparent during the formation of compounds, (ii) pairs of opaque compounds (*n* = 10 pairs), where phonological, orthographic or both changes were present on the compound constituents during the word-formation process, (iii) pairs of legal pseudo-compounds (*n* = 10 pairs). which were formed by two real words, but their combination was a non-word. Pairs of compounds across the three above categories shared a common stem for helping generalization of learning within members of the same word-family. The teacher dictated to students the pairs of compounds. Students had to write down one compound to one column and the other compound to the other column of an A4 sheet. Every accurate spelling of compound was assigned 1 point and every wrong one was given 0 points. The items are given in Appendix II in Supplementary Material along with their word frequencies.

#### (3) Meaning of Compounds (*N* = 16 Items)

The experiment aimed to examine the semantic understanding of compounds. The task entailed 16 single compounds. Children were asked individually by the investigator about the meaning of each item, as follows: “*Could you, please, tell me what the word* ‘πιατοθήκη’ /*piatothiki*/ *dish-rack means*?” Every accurate response in terms of word meaning was assigned 1 point and every inaccurate response was given 0 points. The items are given along with their frequencies in Appendix III in Supplementary Material.

Children’s responses were categorized in terms of their ability to explain etymologically—from a synchronic perspective—the meaning of the compound words as follows (Tsesmeli and Koutselaki, 2013):

(1) (Etymology+): Every answer entailing the morphological constituents of the target word and had an accurate meaning; (2) (Semantics+): Every answer without entailing the morphological constituents of the target word and had an accurate meaning; (3) (Etymology–): Every answer entailing the morphological constituents of the target word and had a wrong meaning; (4) (Semantics–): Every answer without entailing the morphological constituents of the target word and had a wrong meaning. An example of pupils’ responses per each category is given in Appendix IV in Supplementary Material.

### The Intervention Study

The experimental design of the study extends earlier studies by Tsesmeli and Seymour (2009) on English-speaking students and by Tsesmeli (2010) (see also, Tsesmeli and Tsirozi, 2015) on Greek-speaking children. These studies assessed acquisition of spelling either on derivations or on both inflections and derivations by typical and non-typical students who followed advanced stages of schooling. The experimental stimuli of this study are described in detail in the next section.

### Experimental Stimuli

The study was based on the word-pair paradigm which was first introduced by Derwing (1976) as a way of evaluating the word relatedness in terms of meaning. In this study, each pair consisted of two compounds sharing a common stem and was used as an index of the application of morphological strategies in spelling. The items included in the Spelling task (*N* = 60 items) and the Meaning task (*N* = 16 items) were relevant with children’s developmental stage of literacy and vocabulary acquisition in each case (see for the full description of items in section “Experimental tasks, 2 and 3”). For this reason, only semantically transparent compounds of two-constituents were included in the study, and the two constituents were words of Modern Greek. The mean word length of the compounds used in the Spelling task was 10.66 (compounds A: 10.33, compounds B: 11, see Appendix II in Supplementary Material), while the mean word length of the compounds in the Meaning task was 11.37.

For the purposes of the training study, the Spelling task was divided into two subsets of items: (i) Trained items (*n* = 40 items) entailed all the items used in the training program. Items were equally divided into pairs of compounds. Trained items were equally allocated to transparent and opaque items (ii) Untrained items (*n* = 20 items) consisted of pseudo-compounds which did not receive any instruction during the intervention study. These items, however, bear common stems with the relevant compounds, in order to facilitate transfer of learning effects from trained to untrained items.

The Meaning task entailed only compounds, which were a part of the lexical items of the Spelling task used for the training study. Generalization effects could not be evaluated as in Spelling, due to pseudo-compounds, since it is not always possible to define with precision the meaning of pseudo-compounds.

### General Procedure of the Intervention Study

**Table 2** presents the general procedure of the intervention study. Each study included two pre-tests (Spelling task and Meaning task), a training program and two post-tests (Spelling task and Meaning task). The procedure of the study lasted nine sessions for every grade, and was implemented by the investigator in the spring semester of the school year over a period of 3 months.

**Table 2:** General procedure of the intervention study.

***Pre-tests***	
1. Spelling compounds (*N* = 60 items)	G1, G2 experimental/ control
2. Meaning compounds (*N* = 16 items)	G1, G2 experimental
***Intervention***	
1. Training program (5 sessions × 2 h) (*N* = 56 compounds, 10 word families)	G1, G2 experimental
***Post-tests***	
1. Spelling compounds (*N* = 60 items)	G1, G2 experimental/ control
2. Meaning compounds (*N* = 16 pairs)	G1, G2 experimental


### Assessments before and after the Teaching Program

All the items for each task (Spelling task and Meaning task) of the study were randomized to form the pre- and post-tests. The Spelling task was given to experimental and control groups by their teachers to the whole classrooms and lasted two sessions of about 40 min. Both compound words were instructed to dictation as a pair. The students had to write down the spellings on two A4 sheets marked with two separate columns, placing the one compound in the left column and the other compound in the right column.

The Meaning tasks were given individually to the student from each group (except the post-tests for the control groups, due to practical circumstances in terms of school access) by the investigator and lasted two sessions of about 40 min. Each child had to say the meaning of the word and the investigator wrote down their responses on a piece of paper. Children’s definitions of compounds were scholastically written down by the investigator on a paper.

### Training Program

The training program was implemented by the investigator in five sessions of about 2 h for each experimental group (10 h × 2 experimental groups). These separate teaching units involved instruction on five or six compounds which were assigned to a particular word-family (10 word-families). These families were present in children’s school books. Each base word of the family was a phonologically opaque word, so as to permit spelling via the use of a morphological strategy, e.g., φύλλο /*filo*/ *leaf*. This word if spelled phonetically should be written as φίλο /*filo*/ *friend*. In addition, word-families were chosen as the most appropriate items, in order to allow young students to gain insight to every word-family through getting knowledge on the hierarchical relations among its members, and thus, understanding more deeply the way of word-formation and production process (Nunes and Bryant, 2006). Moreover, the training program was based on a systematic progression from transparent items, where the morphemic constituents of the compounds are easily visible to the early readers’ eyes and then to non-transparent items, which are more difficult to be decomposed due to their obscure internal nature. In that way, children could move smoothly from transparent to opaque items, internalizing by practicing the internal structure of compounds using board games.

Hence, the program aimed to teach the students in a step by step way the internal structure of the compounds and how this is related to their spelling and meaning. Each session had a structural and sequential nature and was based on the active participation of the individuals in the classroom settings. More specifically, instruction was targeted toward two main principles: (i) word structure – every compound word is composed of two stems and a suffix; (ii) stem consistency – similar stems of the compounds are spelled identically and carry the same meaning. The sessions were divided into phases, referred to as the Segmentation, the Synthesis, the Spelling practice, the Meaning, and the Oral/Written production of compounds. The Segmentation phase included activities where the children had to analyze the compound word into its constituents, while in the Synthesis phase children were asked to blend two free or bound stems to form a compound word. During the Spelling practice, children should write down the stems of a compound word or the compounds themselves, and at the Meaning phase, children should explain the meaning of the compounds. Finally, during the Oral/Written production, children would produce orally or by writing as many compounds as they could, using the same base word as its main stem.

The control groups did not participate in any of the intervention activities of the training program. However, they attended the formal instructional program followed in schools. This involves a broad variety of activities for enhancing literacy (e.g., reading passages, learning to write small passages, exercises on grammar, vocabulary, etc.), but was not focused on compounds like the training study.

### Materials and Procedure

All sessions of the training program were delivered through group work in the classroom. Each experimental group was divided in three to four smaller groups of three to four students each, depending on the class size. Each group was trained in compounding via 34 educational activities which were constructed by the investigator. The children would have to play as a group via a variety of educational activities such as playing with cards, plastic bricks, puzzles, dices, clock indicators, small boxes, or board games. The first sessions involved mostly segmentation and blending activities using real objects (e.g., bricks, cards, etc.), while the rest sessions included activities in spelling, meaning and oral/written production of compounds. In that way, each child could easily identify, via touching, the appropriate morphological constituents of the words, while the color and size of the objects enhanced the salience and clarity of the constituents of the compounds. Moreover, teaching sessions were analyzed with precision after intervention, giving in this way the appropriate feedback to the experimenter to reflect on the investigator’s training and students’ learning behavior. Students seemed to enjoy learning through the variety of board games used in the classroom.

## Results

Every accurate answer on spelling, meaning, and analogy tasks was assigned 1 point and every inaccurate answer 0 points. Mean percentage accuracy rates are used in every statistical analysis described in the following section.

### Performance on Morphological Awareness, Spelling, and Meaning of Compounds

**Table 3** shows accuracy rates for Morphological Awareness (*N* = 24 for Grade 1, *N* = 40 for Grade 2), Spelling (*N* = 60), and Meaning (*N* = 16) of compounds for the experimental and control groups of Grades 1 and 2. The significance of the difference of the means among the groups on analogy, spelling, and meaning tasks was tested by a 2 × 2 × 3 analysis of variance in which Grade (G1 and G2) and Group (Experimental and Control) were between-participants factors and Task (Analogy, Spelling, and Meaning) was a within-participants factor. This verified significant effects for Task [*F*(2,112) = 24.177, *p* < 0.001, $\eta_{p}^{2}$ = 0.302] and Grade [*F*(1,56) = 15.469, *p* < 0.001, $\eta_{p}^{2}$ = 0.216] but not for Group [*F*(1,56) = 2.537, *p* = 0.117, ns, $\eta_{p}^{2}$ = 0.043]. Following a similar ANOVA between the spelling and meaning tasks showed that spelling accuracy scores were significantly lower than the meaning ones (Task: [*F*(1,56) = 30.883, *p* < 0.001, $\eta_{p}^{2}$ = 0.355]), however, the significant interaction Task × Grade [*F*(1,56) = 18.360, *p* < 0.001, $\eta_{p}^{2}$ = 0.247] indicated that this was the case only for the Grade 1. Grade effects were significant [*F*(1,56) = 37.508, *p* < 0.001, $\eta_{p}^{2}$ = 0.401] but not Group effects [*F*(1,56) = 0.563, *p* = 0.456, $\eta_{p}^{2}$ = 0.010]. Correspondingly, a comparable ANOVA between the spelling and analogy tasks showed significant effects for Task [*F*(1,56) = 45.811, *p* < 0.001, $\eta_{p}^{2}$ = 0.450], revealing that spelling scores were significantly lower than the scores on the morphological awareness task, however, the significant interaction Task × Grade [*F*(1,56) = 54.894, *p* < 0.001, $\eta_{p}^{2}$ = 0.495] indicated again that this result was valid only for the first grade students. Grade but not Group effects were significant (Grade: [*F*(1,56) = 15.925, *p* < 0.001, $\eta_{p}^{2}$ = 0.221], Group: [*F*(1,56) = 0.931, *p* = 0.339, $\eta_{p}^{2}$ = 0.016]). Finally, the ANOVA on the meaning and analogy tasks showed non-significant effects for Task [*F*(1,56) = 0.083, *p* = 0.775, $\eta_{p}^{2}$ = 0.001] and Grade [*F*(1,56) = 0.772, *p* = 0.383, $\eta_{p}^{2}$ = 0.014], suggesting comparable performance on the morphological awareness and meaning of compounds by two grades. Group effects were significant [*F*(1,56) = 6.672, *p* < 0.05, $\eta_{p}^{2}$ = 0.106], indicating better analogy scores on behalf of the control groups from both grades.

**Table 3:** Spelling, Meaning, and Analogy tasks.

	Spelling	Meaning	Word Analogy
G1- Experimental group	40.57 (11.34)	57.10 (27.54)	66.03 (17.73)
G1- Control group	34.18 (16.11)	72.18 (13.61)	73.85 (16.91)
G2- Experimental group	69.18 (18.70)	70.54 (13.00)	61.00 (18.34)
G2- Control group	68.81 (15.94)	74.23 (09.59)	74.06 (17.24)


*Accuracy means (%) (standard deviations in parentheses).*

### Training Effects for Spelling Compounds

**Table 4** shows accuracy rates for pre- and post-tests of the intervention study for the groups along with their gain scores on lexical compounds (*N* = 40). The significance of the difference of the means among the groups was tested by a repeated measures 2 × 2 × 2 analysis of variance in which Grade (G1 and G2) and Group (Experimental and Control) were between-participants factors and Time (Pre-test and Post-test) was a within-participants factor. This verified significant effects for Time [*F*(1,56) = 17.711, *p* < 0.001, $\eta_{p}^{2}$ = 0.240], Grade [*F*(1,56) = 36.710, *p* < 0.001, $\eta_{p}^{2}$ = 0.396], and Group [*F*(1,56) = 6.158, *p* < 0.05, $\eta_{p}^{2}$ = 0.099]. Since the interactions Time × Grade [*F*(1,56) = 15.914, *p* < 0.001, $\eta_{p}^{2}$ = 0.221] and Time × Group [*F*(1,56) = 20.163, *p* < 0.001, $\eta_{p}^{2}$ = 0.265] were significant, further analyses were followed to disentangle these effects. In particular, to evaluate differences between the experimental and control group of Grade 1, a 2 (G1-Experimental and G1-Control) × 2 (Pre- and Post-) ANOVA showed significant effects for Time [*F*(1,27) = 23.483, *p* < 0.001, $\eta_{p}^{2}$ = 0.465] and the interaction Time × Group [*F*(1,27) = 5.191, *p* < 0.05, $\eta_{p}^{2}$ = 0.161]. Further pairwise *t*-tests per each group showed that the experimental group of Grade 1 enhanced significantly its spelling of compounds [*t*(13) = -5.472, *p* < 0.001], as an effect of the intervention, while the control group of the same grade did not present any significant change [*t*(14) = -1.710, *p* = 0.109]. On the other hand, a 2 (G2-Experimental and G2-Control) × 2 (Pre- and Post-) ANOVA between the experimental and control group of the Grade 2 showed non-significant effects for Time [*F*(1,29) = 039, *p* = 0.845, $\eta_{p}^{2}$ = 0.001], while the interaction Time × Group [*F*(1,29) = 21.178, *p* < 0.001, $\eta_{p}^{2}$ = 0.422] was significant. Pairwise *t*-tests per each group showed that the experimental group of Grade 2 presented a significant improvement [*t*(14) = -3.942, *p* < 0.01], due to the intervention, while the control group of the same grade presented a significant fall of its performance [*t*(15) = 2.817, *p* < 0.05]. Finally, developmental differences were also significant between experimental groups of Grades 1 and 2 [*F*(1,27) = 13.077, *p* < 0.01, $\eta_{p}^{2}$ = 0.326] and between control groups of the same grades [*F*(1,29) = 25.073, *p* < 0.001, $\eta_{p}^{2}$ = 0.464]. To summarize, these results showed that the two experimental groups improved significantly their spelling performance on trained compounds due to their participation in the training program, while the two control groups did not improve significantly their performance on these items.

**Table 4:** Spelling lexical compounds.

	Pre-test	Post-test	Gains
G1- Experimental group	40.89 (12.92)	58.92 (17.61)	18.03
G1- Control group	33.00 (15.21)	39.50 (17.40)	6.50
G2- Experimental group	68.83 (20.52)	76.83 (18.95)	8.00
G2- Control group	68.90 (17.02)	61.56 (19.23)	-7.34


*Accuracy means (%) (standard deviations in parentheses).*

### Training Effects for Spelling Compounds in Terms of Transparency

**FIGURE 1:**
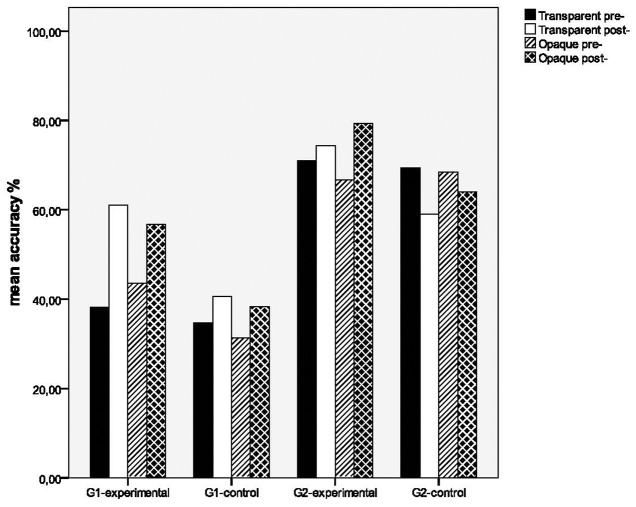
Transparency effects on lexical compounds for the experimental and control groups.

**Figure 1** shows accuracy rates for pre- and post-tests of the intervention study for the groups on transparent (*N* = 20) and opaque lexical compounds (*N* = 20). The significance of the difference of the means among the groups was tested by a 2 × 2 × 4 analysis of variance in which Grade (G1 and G2) and Group (Experimental and Control) were between-participants factors and Transparency (Transparent pre-, Transparent post-, Opaque pre-, and Opaque post-) was a within-participants factor. This revealed significant effects for Transparency [*F*(3,168) = 9.341, *p* < 0.001, $\eta_{p}^{2}$ = 0.143], Group [*F*(1,56) = 6.158, *p* < 0.05, $\eta_{p}^{2}$ = 0.099], and Grade [*F*(1,56) = 36.710, *p* < 0.001, $\eta_{p}^{2}$ = 0.396]. The interactions Transparency × Group [*F*(3,168) = 10.684, *p* < 0.001, $\eta_{p}^{2}$ = 0.160] and Transparency × Grade [*F*(3,168) = 10.634, *p* < 0.001, $\eta_{p}^{2}$ = 0.160] were also significant, hence further analyses were followed to reveal these differences.

In particular, for Group effects, first a 2 (G1-Experimental and G1-Control) × 4 (Transparent pre-, Transparent post-, Opaque pre-, and Opaque post-) ANOVA between the experimental and control groups of Grade 1 was pursued. The analysis showed significant training effects for Transparency [*F*(3,81) = 14.469, *p* < 0.001, $\eta_{p}^{2}$ = 0.349], Transparency × Group [*F*(3,81) = 3.993, *p* < 0.05, $\eta_{p}^{2}$ = 0.129], and Group [*F*(1,27) = 6.528, *p* < 0.05, $\eta_{p}^{2}$ = 0.195]. A separate ANOVA only between pre- and post-test scores on transparent items, it was found that training effects was significant [*F*(1,27) = 24.316, *p* < 0.001, $\eta_{p}^{2}$ = 0.474] as well as the interaction Transparency × Group [*F*(1,27) = 8.298, *p* < 0.01, $\eta_{p}^{2}$ = 0.235], while the pairwise *t*-tests per each group showed that the experimental group of Grade 1 enhanced significantly its spelling performance on transparent items, as an effect of training [*t*(13) = -5.223, *p* < 0.001], while the control group did not present any significant change between the two testing points [*t*(14) = -1.535, *p* = 0.147]. Accordingly, a similar ANOVA between pre- and post-test scores on opaque items revealed significant training effects [*F*(1,27) = 12.640, *p* < 0.01, $\eta_{p}^{2}$ = 0.312], while the interaction Transparency × Group [*F*(1,27) = 1.195, *p* = 0.284, $\eta_{p}^{2}$ = 0.042] was not significant. The pairwise *t*-tests per each group showed that experimental group of Grade 1 improved significantly its spelling scores on opaque items, due to training [*t*(13) = -3.509, *p* < 0.01], while the control group did not present any significant change [*t*(14) = -1.659, *p* = 0.119].

Secondly, a 2 (G2-Experimental and G2-Control) × 4 (Transparent pre-, Transparent post-, Opaque pre-, and Opaque post-) ANOVA between the experimental and control groups of Grade 2 was conducted. This showed non-significant effects for Transparency [*F*(3,87) = 2.398, *p* = 0.073, $\eta_{p}^{2}$ = 0.076] and Group [*F*(1,29) = 1.324, *p* = 0.259, $\eta_{p}^{2}$ = 0.044], but not for Transparency × Group [*F*(3,87) = 8.991, *p* < 0.001, $\eta_{p}^{2}$ = 0.237]. A separate ANOVA only between pre- and post-test scores on transparent items, it was found that training effects was not significant [*F*(1,29) = 1.916, *p* = 0.177, $\eta_{p}^{2}$ = 0.062], while the interaction Transparency × Group [*F*(1,29) = 7.326, *p* < 0.05, $\eta_{p}^{2}$ = 0.202] was significant. The pairwise *t*-tests per each group showed that the experimental group of Grade 2 presented a small non-significant change [*t*(14) = -1.214, *p* = 0.245] on transparent items, while the control group exhibited a significant fall between the two testing points [*t*(15) = 2.488, *p* < 0.05]. Accordingly, a similar ANOVA between pre- and post-test scores on opaque items revealed significant training effects [*F*(1,29) = 4.696, *p* < 0.05, $\eta_{p}^{2}$ = 0.139], while the interaction Transparency × Group [*F*(1,29) = 19.836, *p* < 0.001, $\eta_{p}^{2}$ = 0.406] was also significant. The pairwise *t*-tests per each group showed that the experimental group of Grade 2 improved significantly its spelling scores on opaque items, due to training [*t*(14) = -4.750, *p* < 0.001], while the control group did not present any significant change [*t*(15) = 1.600, *p* = 0.130]. Developmental differences were apparent between the experimental groups of Grades 1 and 2 [*F*(1,27) = 13.077, *p* < 0.01, $\eta_{p}^{2}$ = 0.326] and between the control groups of the same grades [*F*(1,29) = 25.073, *p* < 0.001, $\eta_{p}^{2}$ = 0.464]. To conclude, the above results showed that the experimental group of Grade 1 improved significantly its spelling performance on transparent and opaque compounds due to the intervention, while the experimental group of Grade 2 enhanced significantly its performance only on spelling opaque items. By contrast, the two control groups did not improve their performance on spelling these items.

### Training Effects for Meaning Lexical Compounds

**Table 5** shows accuracy rates for pre- and post-tests of the intervention study for the two experimental groups along with their gain scores on total items (*N* = 16 items). The significance of the difference of the means between the two experimental groups was tested by a 2 × 2 ANOVA in which Grade (G1-Experimental and G2-Experimental) was a between-participants factor and Time (Pre-test and Post-test) was a within-participants factor. This verified significant effects for Time [*F*(1,27) = 46.209, *p* < 0.001, $\eta_{p}^{2}$ = 0.631], while the interaction Time × Grade [*F*(1,27) = 0.724, *p* = 0.402, $\eta_{p}^{2}$ = 0.026] was not significant. In particular, pairwise *t*-tests per each group showed that the experimental group of Grade 1 [*t*(13) = -3.998, *p* < 0.01], as well as the experimental group of Grade 2 [*t*(14) = -8.008, *p* < 0.001] enhanced significantly their meaning performance, as an effect of the intervention. Group effects [*F*(1,27) = 4.888, *p* < 0.05, $\eta_{p}^{2}$ = 0.153] were significant, verifying that Grade 2 had higher accuracy scores at both testing points than Grade 1.

**Table 5:** Meaning of lexical compounds.

	Pre-test	Post-test	Gains
G1- Experimental group	57.10 (27.52)	84.80 (8.73)	27.70
G2- Experimental group	70.54 (13.00)	92.05 (6.01)	21.51


*Accuracy means (%) (standard deviations in parentheses).*

### Qualitative Analysis of Meaning Scores

In the next section, in order to explore further how far the experimental groups would understand the compound structure, children’s data from the meaning task were categorized across four categories in terms of whether they use etymological information at their definitions of these words (see “Meaning of Compounds”). **Table 6** shows this performance of the two groups on the semantic categories in terms of etymology. The significance of the difference of the means among the meaning categories on the accurate responses of the two groups was tested by an ANOVA in which the Meaning category on positive answers (Pre-Etymology+, Post-Etymology+, Pre-Semantics+, and Post-Semantics+) was a within-participants factor and Grade (G1-experimental and G2-experimental) was a between-participants factor. The analysis showed significant effects for the Meaning categories [*F*(3,81) = 97.829, *p* < 0.001, $\eta_{p}^{2}$ = 0.784], while the interaction Categories × Grade [*F*(3,81) = 13.881, *p* < 0.001, $\eta_{p}^{2}$ = 0.340] and Grade [*F*(1,27) = 4.340, *p* < 0.05, $\eta_{p}^{2}$ = 0.138] were also significant, indicating that the two grades improved significantly their performance on the Etymology and Semantics category, as an effect of the intervention. Accordingly, the significance of the difference of the means among the categories on the inaccurate responses of the same groups was also tested by a similar analysis of variance in which the Meaning category on negative answers (Pre-Etymology-, Post-Etymology-, Pre-Semantics-, and Post-Semantics-) was a within-participants factor. The analysis showed significant training effects for the Meaning categories [*F*(3,81) = 11.062, *p* < 0.001, $\eta_{p}^{2}$ = 0.291], while the interaction Categories × Grade [*F*(3,81) = 3.534, *p* < 0.05, $\eta_{p}^{2}$ = 0.116] and Grade [*F*(1,27) = 4.340, *p* < 0.05, $\eta_{p}^{2}$ = 0.138] were also significant, indicating that the two grades decreased significantly their meaning errors due to the intervention. The above results verified that both experimental grades increased substantially their accurate etymological responses after intervention, with a parallel decrease of the relevant errors, thus suggesting a considerable improvement at their understanding of the morphological structure of the compounds.

**Table 6:** Meaning categories (%).

	G1- Experimental	G2- Experimental
Etymology+ pre-	27.07 (12.89)	34.32 (11.66)
post-	74.55 (14.80)	78.33 (11.29)
gains	47.48	44.01
Semantics+ pre-	03.17 (3.20)	38.51 (7.11)
post-	12.05 (13.07)	15.83 (10.59)
gains	8.88	-22.68
Etymology- pre-	12.16 (12.44)	11.11 (8.51)
post-	11.60 (8.44)	5.41 (3.99)
gains	-0.56	-5.7
Semantics- pre-	5.02 (7.35)	16.04 (7.62)
post-	1.78 (3.82)	0.00 (0.000)
gains	-3.24	-16.04


*(Standard deviations in parentheses)*.

### Training Effects on Spelling and Meaning Lexical Compounds

**FIGURE 2:**
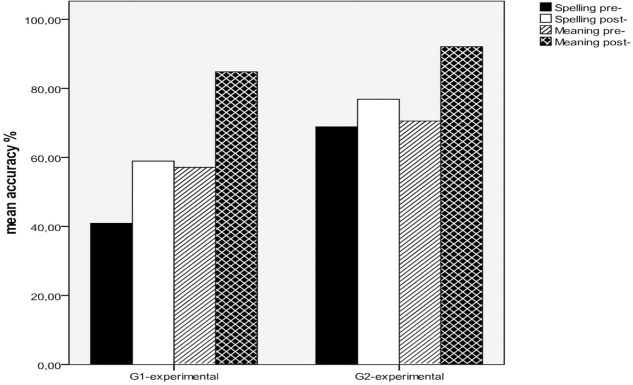
Spelling and Meaning training effects on lexical compounds for the experimental groups.

**Figure 2** illustrates the spelling and meaning performance of the two experimental groups on the lexical compounds for the pre- and post-tests of the intervention study, since the two tasks share partly identical items (e.g., πιατοθήκη [*piatothiki*-dishrack]) and partly items with a common morpheme (λαχαν*ό*φυλλο [*lachanofilo*-cabbage-leaf] for spelling, μαρουλ*ό*φυλλο [*marulofilo*-lettuce-leaf] for meaning) (see, Appendices II and III in Supplementary Material). The significance of the difference of the means between the spelling and meaning of the compounds was tested by a 2 × 2 × 2 ANOVA in which Grade (G1-experimental and G2-experimental) was a between-participants factor and the Time (Pre- and Post-) and the Task (Spelling and Meaning) were the two within-participants factors. This verified significant effects for Time [*F*(1,27) = 84.956, *p* < 0.001, $\eta_{p}^{2}$ = 0.759], while the interaction Time × Grade was marginally non-significant [*F*(1,27) = 3.935, *p* = 0.058, $\eta_{p}^{2}$ = 0.127], indicating significant training effects for the two tasks. Significant effects were revealed also for the Task [*F*(1,27) = 17.777, *p* < 0.001, $\eta_{p}^{2}$ = 0.397], while the interaction Task × Grade [*F*(1,27) = 14.727, *p* < 0.01, $\eta_{p}^{2}$ = 0.353] was not significant, indicating that meaning performance was significantly higher than spelling for both experimental groups. The interaction Time × Task was also significant [*F*(1,27) = 7.997, *p* < 0.01, EQUATION 0.229], suggesting that training effects were stronger for meaning than for spelling for both experimental groups as indexed by the non-significant interaction Time × Task × Group [*F*(1,27) = 0.223, *p* = 0.641, EQUATION 0.008]. Developmental effects in favor of Grade 2 were present as shown by the significant Group effect [*F*(1,27) = 14.727, *p* < 0.01, EQUATION 0.353].

### Generalization Effects for Spelling Pseudo-Compounds

The generalization of learning was tested by comparing pre- and post-test results for the untrained pseudo-compounds who bear, however, similar bound stems with the trained ones, to facilitate transfer of learning from trained to untrained items. **Table 7** shows accuracy rates for pre- and post-tests of the intervention study for the groups along with their gain scores on untrained pseudo-compounds (*N* = 20). The significance of the difference among the groups was tested by a 2 × 2 × 2 analysis of variance in which Grade (G1 and G2) and Group (Experimental and Control) were between-participants factors and Time (Pre-test and Post-test) was a within-participants factor. This verified significant effects for Time [*F*(1,56) = 4.154, *p* < 0.05, $\eta_{p}^{2}$ = 0.069] and Grade [*F*(1,56) = 40.990, *p* < 0.001, $\eta_{p}^{2}$ = 0.423], but not for Group [*F*(1,56) = 1.786, *p* = 0.187, $\eta_{p}^{2}$ = 0.031]. The interaction Time × Group [*F*(1,56) = 3.045, *p* = 0.086, $\eta_{p}^{2}$ = 0.052] was not significant. Since only the interaction Time × Grade was significant [*F*(1,56) = 4.378, *p* < 0.05, $\eta_{p}^{2}$ = 0.073], two separate 2 (G1-experimental and G2-experimental) × 2 (Pre- and Post-) and 2 (G1-control and G2-Control) × 2 (Pre- and Post-) ANOVAs were performed. The first one between the two experimental groups showed significant effects for Time [*F*(1,27) = 10.660, *p* < 0.01, $\eta_{p}^{2}$ = 0.283] and the interaction Time × Group [*F*(1,27) = 6.328, *p* < 0.05, $\eta_{p}^{2}$ = 0.190] indicating significant generalization effects which differed between the two experimental groups. Hence, the pairwise *t*-tests on these two groups showed that the experimental Grade 1 generalized significantly to untrained pseudo-compounds [*t*(13) = -3.229, *p* < 0.01], while the experimental Grade 2 did not achieve to generalize to the non-lexical items [*t*(14) = -0.770, *p* = 0.454]. Accordingly, the second analysis between the two control groups showed non-significant generalization effects [*F*(1,29) = 0.033, *p* = 0.856, $\eta_{p}^{2}$ = 0.001] for both groups as indexed by the non-significant interaction Time × Grade [*F*(1,29) = 0.629, *p* = 0.434, $\eta_{p}^{2}$ = 0.021]. Developmental effects in terms of spelling pseudo-compounds were significant for both experimental [*F*(1,27) = 18.507, *p* < 0.001, $\eta_{p}^{2}$ = 0.407] and control groups [*F*(1,29) = 22.833, *p* < 0.001, $\eta_{p}^{2}$ = 0.441], suggesting that first graders spelled better these items than the second graders. As a conclusion, the main outcome is that only the experimental group of Grade 1 generalized significantly its spelling performance on untrained pseudo-compounds due to the intervention, while the experimental group of Grade 2 and the two control groups did not improve their performance on spelling these items.

**Table 7:** Spelling pseudo-compounds.

	Pre-test	Post-test	Gains
G1- Experimental group	40.00 (12.55)	52.85 (17.39)	12.85
G1- Control group	36.66 (20.32)	39.66 (21.16)	3.00
G2- Experimental group	70.00 (17.11)	71.66 (17.79)	1.66
G2- Control group	68.75 (17.17)	66.87 (18.33)	-1.88


*Accuracy means (%) (standard deviations in parentheses).*

### Generalization Effects for Spelling Pseudo-Compounds in Terms of Transparency

**FIGURE 3:**
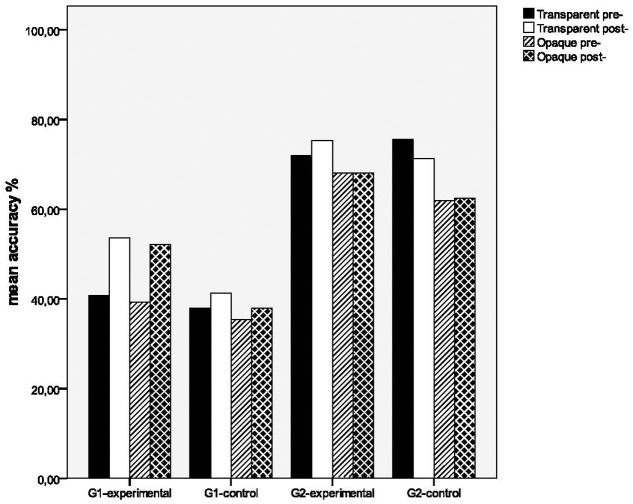
Transparency effects on pseudo-compounds for the experimental and control groups.

**Figure 3** shows accuracy rates for pre- and post-tests of the intervention study for the groups on transparent (*N* = 10) and opaque (*N* = 10) pseudo-compounds. The significance of the means difference among the groups was tested by a 2 × 2 × 4 analysis of variance in which Grade (G1 and G2) and Group (Experimental and Control) were between-participants factors and Transparency (Transparent pre-, Transparent post-, Opaque pre-, and Opaque post-) was a within-participants factor. This verified significant effects for Transparency [*F*(3,168) = 5.682, *p* < 0.01, $\eta_{p}^{2}$ = 0.092] and Grade [*F*(1,56) = 40.990, *p* < 0.001, $\eta_{p}^{2}$ = 0.423], while Group effects [*F*(1,56) = 1.786, *p* = 0.187, $\eta_{p}^{2}$ = 0.031], as well as the interaction Transparency × Group [*F*(3,168) = 1.990, *p* = 0.117, $\eta_{p}^{2}$ = 0.034] were not significant. Since the interaction Transparency × Grade [*F*(3,168) = 3.369, *p* < 0.05, $\eta_{p}^{2}$ = 0.057] was significant, further separate for each grade 2 × 4 ANOVAs were performed. In detail, the first one between the two experimental groups of Grades 1 and 2 showed significant generalization effects for Transparency [*F*(3,81) = 4.665, *p* < 0.01, $\eta_{p}^{2}$ = 0.147], while the interaction Transparency × Grade [*F*(3,81) = 2.579, *p* = 0.059, $\eta_{p}^{2}$ = 0.087] was marginally significant. An exploration of these differences via the pairwise *t*-tests per each group showed that the experimental group of Grade 1 generalized significantly its spelling performance to transparent [*t*(13) = -2.857, *p* < 0.05] and opaque pseudo-compounds [*t*(13) = -2.482, *p* < 0.05], while the experimental group of Grade 2 did not present any generalization effects neither to transparent [*t*(14) = -1.160, *p* = 0.265] nor to opaque items [*t*(14) = 0.000, *p* = 1.000]. Besides, the second ANOVA between the two control groups of Grades 1 and 2 showed significant generalization effects for Transparency [*F*(3,87) = 3.177, *p* < 0.05, $\eta_{p}^{2}$ = 0.099], while the interaction Transparency × Grade [*F*(3,87) = 1.518, *p* = 0.216, $\eta_{p}^{2}$ = 0.050] was not significant. This was also verified by the pairwise *t*-tests that showed that both control groups did not generalize neither to transparent [G1, *t*(14) = -0.564, *p* = 0.582, G2, *t*(15) = 1.000, *p* = 0.333], nor to opaque items [G1, *t*(14) = -0.541, *p* = 0.597, G2, *t*(15) = -0.115, *p* = 0.910]. Grade effects were significant for both experimental [*F*(1,27) = 18.507, *p* < 0.001, $\eta_{p}^{2}$ = 0.407] and control groups [*F*(1,29) = 22.833, *p* < 0.001, $\eta_{p}^{2}$ = 0.441], indicating developmental differences in the accuracy spelling scores on pseudo-compounds between the groups. To finalize, these results showed that the experimental group of Grade 1 generalized significantly its spelling on transparent and opaque pseudo-compounds due to the intervention, but the experimental group of Grade 2 and the two control groups did not show generalization effects to any of these items.

## Discussion

The study aimed to evaluate the intervention effects on spelling and semantic understanding of compounds by Greek students in their first years of schooling via a rich variety of educational activities in classroom settings. The intervention study extends earlier case-studies by Tsesmeli and Seymour (2009) on English-speaking students and Tsesmeli (2010; Tsesmeli and Tsirozi, 2015) on Greek-speaking students who followed advanced grades of schooling on inflectional and derivational items, and confirmed the main hypotheses and aims of the study.

Before the commencement of the study, findings from the experimental tasks on spelling, meaning and morphological awareness showed that first grade students exhibited lower performance on spelling compounds than on their semantic understanding, while their ability to produce new compounds based on given ones via analogy was at the same level with their meaning scores. The three tasks did not differ significantly for the second graders. Developmental differences on experimental groups were evident between first and second graders only for spelling (40.57% vs. 69.18%), while for meaning (57.10% vs. 70.54%) and morphological awareness of compounds (both over 60%), differences between the grades were not significant. These results are in line with another study’s findings in relation to compounds by Tsesmeli and Koutselaki (2013) on typically developing Greek students throughout schooling, that showed that semantic understanding of words significantly precedes spelling of words.

Findings from the intervention study on spelling lexical compounds showed that the two experimental groups following the first and second grades was enhanced their spelling performance considerably after the intervention, due to their participation in the training program. In particular, students of the experimental group of Grade 1 improved their mean spelling scores on morphologically complex words up to 58.92% (gains: 18.03) during a short program of 10 h over 2 week’s via enjoyable classroom activities. The main training effect was significant, indicating that the change of the experimental group of Grade 1 due to training was significantly higher, if compared with their control group of the same grade. On the other hand, the experimental group of Grade 2 students presented a change, which was much smaller than the relevant group of Grade 1 students (up to 76.83%, gains: 8.00), possibly due to ceiling effects. Change of Grade 2 was also a significant one in comparison with their control group, which exhibited an opposing pattern, since it decreased significantly its performance, possibly due to lack of motivation at the re-test session. Developmental effects on spelling accuracy levels were significant for both the experimental and the control groups, as expected. These results are in line with experimental literature (Nunes and Bryant, 2006; Tsesmeli and Seymour, 2009; Bowers et al., 2010) extending findings from children at later stages of literacy but on other types of morphologically complex words, i.e., inflections and derivations. Moreover, they corroborate relevant data from other intervention studies on early readers, such as kindergarteners and first and second graders (Casalis and Colé, 2009; Apel and Diehm, 2014; Ramirez et al., 2014; Wolter and Dilworth, 2014; Lyster et al., 2016) and lead to a confident conclusion that it is feasible to train children at a very early age in such complex and demanding vocabulary, as the compounds, in the natural environment of a common school class.

A notable query here is how spelling performance is varied among different types of items in terms of morphological transparency. The data from lexical compounds showed that, before the beginning of the study, first grade students from the experimental group had about the same performance on transparent (38.21%) and opaque (43.57%) compounds, however, after intervention, they attempted to increase significantly both types of items, with the increase being more profound on transparent items (gains: 22.86 vs. 13.21 for opaque items). The contrasting profile was shown for second grade students from the experimental group. In particular, before the beginning of the study, while they had, as first graders, comparable performance on transparent (71%) and opaque (66.66%) compounds, they attempted to present a significant change only on opaque items (gains: 12.67 vs. 3.33, transparent items) after training. On the contrary, neither control groups showed any significant improvement either on transparent or on opaque compounds. These unexpected findings indicate weak transparency effects prior intervention, contrary to experimental evidence (Kuo and Anderson, 2006; Goodwin and Ahn, 2013; Goodwin et al., 2014), however, first and second grade young readers attempted to make significant gains either on transparent or opaque items accordingly, after internalizing word-formation rules during training.

These results are not easily interpretable, however, it can be hypothesized that the choice of task stimuli might affected spelling performance. In particular, both transparent and opaque compounds were chosen to belong to the same word family, which means that they had a common constituent (the base word of the family), in order to facilitate progressive learning from transparent to non-transparent items by young readers. This might resulted to comparable spellings (e.g., transparent: ‘λαχαν*ό*φυλλο’ [*lachanofilo*-cabbage-leaf] vs. opaque: αμπελ*ό*φυλλο [*abelofilo*-vine-leaf], see Appendix II in Supplementary Material for their constituents) that evoked comparable spelling scores. In addition, the degree of transparency in these sets of compounds is much smaller in comparison with compounds entailing bound morphemes from Ancient Greek (see, Appendix I in Supplementary Material) where its internal structure is really obscured and it is not easily discernable by a young reader. These words were not chosen to be stimuli of an early intervention as not at all appropriate for very young readers. However, in the later case, the transparency effects on spelling would be expected to be strong, as indicated by Greek data on older students (Tsesmeli, 2008; Rousoulioti, 2011). In any case, further research is needed for this important aspect of compounding, along with other psycholinguistic features such as semantic transparency, compound frequency and prosody, as shown in recent literature (Lemhöfer et al., 2011; Koester, 2014).

Another important issue that should be addressed at this point, is training effects in relation to semantic understanding of morphologically complex items. In particular, although both experimental groups had a different starting point before intervention (57% vs. 70%), they attempted to present significant gains of important size (27.50 vs. 21.51), and this effect was stronger for the early beginners of the first grade. Further comparisons between spelling and meaning scores showed that training effects for meaning were significantly stronger than relevant effects for spelling, and this was the case for both first and second graders. This finding is particularly important, indicating that morphological awareness training on very young readers even from their first years of schooling can lead to significant increments not only to literacy skills, such as spelling (Nunes and Bryant, 2006; Tsesmeli and Seymour, 2009; Tsesmeli, 2010), but also to vocabulary improvement, forming the basis for later improvements on reading comprehension and academic success (Ramirez et al., 2014).

The above results are enlightened by the qualitative categorization of students’ responses on meaning. In particular, a careful examination of pre-test responses in relation to compounds’ explanations indicated that the correct etymological answers were multiplied rather than the correct non-etymological ones for both experimental groups. Most considerably, experimental groups attempted to increase substantially their etymological positive responses after the intervention (gains: 47.48 vs. 44.01), and at the same time to decrease noticeably their etymological and semantic errors, indicating strongly that children participating in the program were able to use considerably more often the etymological information to convey the meaning of the compounds, and the way they are spelled. Namely, it was shown for both experimental groups that children recognized the morphological constituents of the target word prior intervention to a larger extent and this effect was highly accelerated as a means of intervention. This may suggest that this type of awareness exists in children’s brains and regardless of its small size it can be used as a morphological strategy to improve their vocabulary and spelling performance (Tsesmeli and Koutselaki, 2013). Also, according to Ramirez et al. (2014), morphological awareness is “generative,” thus children can use it independently to analyze thousands of words and derive meanings for new items.

The postulation at this point is that children who developed better skills of morphological awareness may have a benefit in attaining a complex vocabulary, as suggested by Bowers and Kirby (2010), who showed that students’ skill to recognize bases in complex words contributed an important amount of variance to their comprehension, and hence predicted their general vocabulary knowledge. This can lead to better reading comprehension skills, as shown by the remarkable study by Lyster et al. (2016), where children who received morphological training at the pre-school exhibited better skills in this aspect 6 years later, at Grade 6, than the control group. These results may be broadened by recent evidence in the context of morphological processing by Feldman et al. (2015) who demonstrated that meaning influences even the very early stages of visual recognition of morphemes, suggesting an early and dynamic interaction of meaning and form. This was based on the data tracking the earliest possible time course of processing, where the facilitation based on the form of a shared morpheme (*sneaker-sneak*) is weaker than facilitation based on semantic similarity along with its form (*sneaky-sneak*) (p. 167), although this topic is still controversial, since models of visual word recognition, as well as current studies, suppose that the form of a word is accessed before its meaning (Pylkkänen et al., 2004; Goucha and Friederici, 2015).

According to Seymour (1998), an important issue in the assessment of the intervention studies relates to whether children would be able to generalize learning from instructed items to uninstructed ones. Generalization is further assisted via homologous items of common word structure and identical lexical units, as in this study. In particular, findings showed that first grade students, who took part in the program, attempted to generalize the pseudo-compounds to a considerable degree (gains: 12.85), while the relevant control group did not show any significant generalization change, confirming that generalization effects by early readers was due to training. Unfortunately, this was not the case, for the experimental group of second grade whose change was small and did not reach significance, while there was not any significant change for its relevant control group. Findings in terms of generalization effects in relation to transparency indicated different profiles for the two experimental groups. In particular, early beginners of first grade showed significant generalization effects of equal size both on transparent and opaque pseudo-compounds (gains: 12.86), while they had a similar performance on these items before intervention. Late beginners of second grade showed small and non-significant generalization effects only on transparent pseudo-compounds, while none of the controls presented any significant generalization effects on transparent or opaque items. However, the results are positive, at least for the first graders, suggesting that even very young readers of this age are able to make use of their instructed knowledge to spell new items and in consistency with related studies, as by Nunes and Bryant (2006) and Tsesmeli and Seymour (2009) (see also, Tsesmeli, 2010; Tsesmeli and Tsirozi, 2015) where older children transferred principle learning to derivational items of similar word structure.

Therefore, the results of this study have a number of substantial implications for educational practice. The main suggestion is that morphological awareness is of importance and that instruction of the morphological structure of the words would be valuable to students in regular classroom settings (Nunes and Bryant, 2006), even from their first years of schooling. Students may need to apply in a specific and concrete way the most abstract ideas of internal word structure (Tsesmeli and Tsirozi, 2015). This process could be further assisted via a variety of appropriate educational materials, as used in the present study, such as color-coding techniques, real objects, board-games in small groups, in order to increase active involvement of the learner to the task. Moreover, comparable interventions should improve the training of the morphemic structure of the words in an explicit and sequential way from the simplest to the most complex structures (i.e., transparent vs. opaque items). Also, spelling should be complemented with the understanding of these forms, since it has been shown that morphological training increases student’s vocabulary development (Bowers and Kirby, 2010; Ramirez et al., 2014; Lyster et al., 2016).

Some of the limitations of this study would be the lack of control group’s data in the meaning study, due to practical circumstances in terms of school access, to facilitate comparison with experimental groups, as in the spelling study. However, significant results from the meaning study suggest that the improvement in meaning scores was due to training. Moreover, the investigation of the variability of the students in terms of ability levels would add important aspects to the present findings, since there is a number of individuals that might differ in their cognitive ability and degree of improvement, as in the study by Ramirez et al. (2014). Besides, one significant query for research in the future would be the examination of the acquisition of morphologically complex forms and their response to more extensive treatment in larger samples for the experimental and control groups, along with other cognitive and psycholinguistic factors (i.e., general intelligence, reading comprehension, standardized measures of reading/spelling, etc.) in order to enhance the generalization of the results for the relevant populations (Apel and Diehm, 2014; Wolter and Dilworth, 2014). Finally, an important factor that should be taken into account is the duration of the intervention which was short (five sessions, 10 h) in order to produce stronger training and generalization effects for the experimental groups.

However, this study is among the first attempts at morphographic training in compounding in Greek, which is a phonologically transparent orthography (Seymour et al., 2003) but with a rich morphology (Ralli, 2013; Babiniotis, 2016). The outcome indicated that intervention was efficient in improving the spelling and semantic understanding of morphologically complex words for early readers of the first years of schooling. These results are consistent with experimental literature (Nunes and Bryant, 2006; Bowers et al., 2010; Apel and Diehm, 2014; Ramirez et al., 2014; Wolter and Dilworth, 2014; Lyster et al., 2016) and are especially valuable for the development of complementary approaches to the educational interventions of typically developing children in regular classroom settings.

## Ethics Statement

This study was carried out in accordance with the recommendations of ethical guidelines of Greek Psychological Society with written informed consent from all subjects. All subjects gave written informed consent in accordance with the Declaration of Helsinki. The protocol was approved by the regional educational authority of schools.

## Author Contributions

ST conceptualized the research project, supervised the data-collection, run the statistics, conducted the writing of the paper and the interpretation of the findings.

## Conflict of Interest Statement

The author declares that the research was conducted in the absence of any commercial or financial relationships that could be construed as a potential conflict of interest.

## Footnotes

1 Both experimental and control groups entailed students of a wide range of scores in terms of reading, spelling and vocabulary skills. Variation in terms of centiles for reading words (RW): Experimental group: 8/14: 100°–50°, 6/14: 40°, Control group: 14/15: 100°–50°, 1/14: 30°. For reading non-words (RNW): Experimental group: 4/14: 70°–40°, 5/14: 30°, 5/14: 20°–10°. Control group: 10/15: 70°–40°, 3/15: 30°, 2/15: 20°. For spelling words (SW): Experimental group: 11/15: 90°–70°, 4/15: 25°, Control group: 15/16: 95°–70°, 1/15: 25°. For written vocabulary: Experimental group: 8/15: 75°–40°, 7/15: < 25°, Control group: 12/16: 98°–60°, 4/6: 40°. RW/RNW/SW centiles are based on a sample of about 600 children (Padeliadu and Sideridis, 2000; Mouzaki et al., 2007). Vocabulary centiles are based on a sample of about 2.500 children (Tafa, 1995).
