# Supplementary material for: Spelling and Meaning of Compounds in the Early School Years through Classroom Games: An Intervention Study
Source: Front Psychol. 2017 Nov 29;8:2071. doi: 10.3389/fpsyg.2017.02071 (PMC5712978; doi:10.3389/fpsyg.2017.02071)
Supplement: Supplementary file 1 [file Table_1.PDF]

frontiers

Supplementary Material

Spelling and meaning of compounds in the early school years through classroom games: an intervention study

Styliani N. Tsesmeli[1]

[1] Psychology Section, Department of Primary Education, University of Patras, Rion, Greece.

*** Correspondence:**
e-mail: stsesmeli@upatras.gr

Appendix I

Table 1. Examples of the Analogy Task (A:B/ C:D)

	A	*B*	*C*	*D*
TRANSPARENT compounds (B, D)	κόκκινο<br>/kokino/<br>red	χρυσοκόκκινο<br>/chrisokokino/<br>gold-red	πράσινο<br>/prasino/<br>green	χρυσοπράσινο<br>/chrisoprasino/<br>gold-green
OPAQUE compounds (B, D)	μικρός<br>/mikros/<br>small	μικρόσωμος<br>/mikrosomos/<br>with a small body	μεγάλος<br>/meɣalos/<br>large	μεγαλόσωμος<br>/meɣalosomos/<br>with a large body
Compounds with BOUNT morphemes of Ancient Greek origin (B, D)	καπνός<br>/kapnos/<br>smoke	καπνοδόχος<br>/kapnoðochos/<br>chimney	ξένος<br>/xenos/<br>foreign/guest	ξενοδόχος<br>/xenoðochos/<br>guest-house owner
PREFIXED compounds (B,D)	φέρνω<br>/ferno/<br>bring	καταφέρνω<br>/kataferno/<br>achieve	φθάνω<br>/fthano/<br>arrive	καταφθάνω<br>/katafthano/<br>arrive unexpectedly

Note: A, C are simple words and one of the constituents of the C & D compounds. D compounds are children's expected answers.

Appendix II

Table 1. Spelling compounds (pre- & post-test of the Intervention study) (N=60 items)

		f	Compound A	Compound A: 1st stem	Compound A: 2nd stem	f	Compound B	Compound B: 1st stem	Compound B: 2nd stem
T	TR	0	λαχανόφυλλο<br>*/lachanofilo/*<br>cabbage-leaf	λάχανο<br>*/lachano/*<br>cabbage	φύλλο<br>*/filo/*<br>leaf	128	τριαντάφυλλο<br>/triantafilo/<br>rose	τριάντα<br>/trianta/<br>thirty	φύλλο<br>/filo/<br>leaf
T	TR	13	ζωοτροφή<br>/zootrofi/<br>animal food	ζώο<br>/zoo/<br>animal	τροφή<br>/trofi/<br>food	6	ζωοκλοπή<br>/zooklopi/<br>animal-theft	ζώο<br>/zoo/<br>animal	κλοπή<br>/klopi/<br>theft
T	TR	2	ξυλόσομπα<br>/xilosoba/<br>wood heater	ξύλο<br>/xilo/<br>wood	σόμπα<br>/soba/<br>heater	0	ξυλομπογιά<br>/xiloboɤia/<br>colored pencil	ξύλο<br>/xilo/<br>wood	μπογιά<br>/boɤia/<br>pencil
T	TR	0	βιβλιοκλοπή<br>/vivlioklopi/<br>book-theft	βιβλίο<br>/vivlio/<br>book	κλοπή<br>/klopi/<br>theft	3	βιβλιόφιλος<br>/vivliofilos/<br>friend of books	βιβλίο<br>/vivlio/<br>book	φίλος<br>/filos/<br>friend
T	TR	23	εργαλειοθήκη<br>/erɤaliothiki/<br>tool-kit	εργαλείο<br>/erɤalio/<br>tool	θήκη<br>/thiki/<br>case	0	πιατοθήκη<br>/piatothiki/<br>plate-case	πιάτο<br>/piato/<br>Plate	θήκη<br>/thiki/<br>case
T	TR	69	αυτοκινητόδρομος<br>/aftokinitoðromos/<br>car-road	αυτοκίνητο<br>/aftokinito/<br>car	δρόμος<br>/ðromos/<br>road	0	ποδηλατόδρομος<br>/poðilatoðromos/<br>bicycle-road	ποδήλατο<br>/Poðilato/<br>bicycle	δρόμος<br>/ðromos/<br>road
T	TR	26	μοναχοπαίδι<br>/monachopeði/<br>the only child	μονάχο<br>/monacho/<br>single	παιδί<br>/peði/<br>child	7	στερνοπαίδι<br>/sternopeði/<br>the last child	στερνό<br>/sterno/<br>last	παιδί<br>/peði/<br>child
T	TR	126	εξώπορτα<br>/exoporta/<br>outdoor	έξω<br>/exo/<br>out	πόρτα<br>/porta/<br>door	25	καγκελόπορτα<br>/kageloporta/<br>raildoor	κάγκελο<br>/kagelo/<br>rail	πόρτα<br>/porta/<br>door
T	TR	0	αυγόφετα<br>/avɤofeta/<br>eggslice	αυγό<br>/avɤo/<br>egg	φέτα<br>/feta/<br>slice	0	αυγόφλουδα<br>/avɤofslouða/<br>eggshell	αυγό<br>/avɤo/<br>egg	φλούδα<br>/flouða/<br>shell

T	TR	0	αγριοπούλι<br>/aɤriopouli/<br>wildbird	άγριο<br>/aɤrio/<br>wild	πουλί<br>/pouli/<br>bird	1	μαυροπούλι<br>/mavropouli/<br>blackbird	μαύρο<br>/mavro/<br>black	πουλί<br>/pouli/<br>bird
T	OP	0	αμπελόφυλλο<br>/abelofilo/<br>vine-leaf	αμπέλι<br>/abeli/<br>vine	φύλλο<br>/filo/<br>leaf	2	μαρουλόφυλλο<br>/maroufilo/<br>lettuce-leaf	μαρούλι<br>/marouli/<br>lettuce	φύλλο<br>/filo/<br>leaf
T	OP	0	ζωόφιλος<br>/zoofilos/<br>He who loves the animals	ζώο<br>/zoo/<br>animal	φιλώ<br>/filo/<br>love	2	ζωολόγος<br>/zooloɤos/<br>He who speaks about animals	ζώο<br>/zoo/<br>animal	λέγω<br>/leɤo/<br>speak about
T	OP	1	κουρτινόξυλο<br>/kourtinoxilo/<br>curtain rail	κουρτίνα<br>/kourtina/<br>curtain	ξύλο<br>/xilo/<br>wood	0	ροδόξυλο<br>/roðoxilo/<br>rosewood	ρόδο<br>/roðo/<br>rose	ξύλο<br>/xilo/<br>wood
T	OP	1	βιβλιόσημο<br>/vivliosimo/<br>bookstamp	βιβλίο<br>/vivlio/<br>book	σημείο<br>/simio/<br>sign	179	βιβλιοπωλείο<br>/vivliopolio/<br>bookstore	βιβλίο<br>/vivlio/<br>book	πωλώ<br>/polo/<br>to sell
T	OP	0	μολυβοθήκη<br>/molivothiki/<br>pencilcase	μολύβι<br>/molivi/<br>/pencil/	θήκη<br>/thiki/<br>case	0	σαπουνοθήκη<br>/sapounothiki/<br>soapcase	σαπούνι<br>/sapouni/<br>soap	θήκη<br>/thiki/<br>case
T	OP	6	ποδαρόδρομος<br>/poðaroðromos/<br>pathwalk	ποδάρι<br>/poðari/<br>leg	δρόμος<br>/ðromos/<br>road	0	αμαξόδρομος<br>/amaxoðromos/<br>car-road	αμάξι<br>/amaxi/<br>car	δρόμος<br>/ðromos/<br>road
T	OP	0	λεβεντόπαιδο<br>/leventopeðo/<br>brave man	λεβέντης<br>/levedis/<br>brave	παιδί<br>/peði/<br>child	2	αλητόπαιδο<br>/alitoðo/<br>rake man	αλήτης<br>/alitis/<br>rake	παιδί<br>/peði/<br>child
T	OP	1	αυγολέμονο<br>/avɤolemono/<br>egg-lemon (kind of Greek sauce)	αυγό<br>/avɤo/<br>egg	λεμόνι<br>/lemoni/<br>lemon	0	αυγότσουφλο<br>/avɤotsouflo/<br>eggshell	αυγό<br>/avɤo/<br>egg	τσόφλι<br>/tsofli/<br>shell
T	OP	10	τζαμόπορτα<br>/tzamoporta/<br>glassdoor	τζάμι<br>/tzami/<br>glass	πόρτα<br>/porta/<br>door	21	μπαλκονόπορτα<br>/balkonoporta/<br>French door	μπαλκόνι<br>/balkoni/<br>balcony	πόρτα<br>/porta/<br>door
T	OP	6	θαλασσοπούλι<br>/thalassopouli/<br>seabird	θάλασσα<br>/thalassa/<br>sea	πουλί<br>/pouli/<br>bird	1	νυχτοπούλι<br>/nichtopouli/<br>nightbird	νύχτα<br>/nichta/<br>night	πουλί<br>/pouli/<br>bird
UN	PS	-	ξυλόφυλλο<br>/xilofilo/	ξύλο<br>/xilo/<br>wood	φύλλο<br>/filo/<br>leaf	-	παπουτσόφυλλο<br>/papoutsofilo/	παπούτσι<br>/papoutsi/<br>shoe	φύλλο<br>/filo/<br>leaf

UN	PS	-	ζωότρελλος<br>/zootrelos/	ζώο<br>/zoo/<br>animal	τρελλός<br>/trelos/<br>crazy	-	ζωόμουσος<br>/zoomousos/	ζώο<br>/zoo/<br>animal	μούσα<br>/moussa/<br>muse
UN	PS	-	σαπιόξυλο<br>/sapioxilo/	σάπιο<br>/sapio/<br>rotten	ξύλο<br>/xilo/<br>wood	-	τραπεζόξυλο<br>/trapezoxilo/	τραπέζι<br>/trapezi/<br>table	ξύλο<br>/xilo/<br>wood
UN	PS	-	βιβλιόλεξο<br>/vivliolexo/	βιβλίο<br>/vivlio/<br>book	λέξη<br>/lexi/<br>word	-	βιβλιοπερίπτερο<br>/vivlioperiptero/	βιβλίο<br>/vivlio/<br>book	περίπτερο<br>/periptero/<br>kiosk
UN	PS	-	συννεφοθήκη<br>/sinefothiki/	σύννεφο<br>/sinefo/<br>cloud	θήκη<br>/thiki/<br>case	-	μελισσοθήκη<br>/melisothiki/	μέλισσα<br>/melissa/<br>bee	θήκη<br>/thiki/<br>case
UN	PS	-	φτερόδρομος<br>/fteroðromos/	φτερό<br>/ftero/<br>feather	δρόμος<br>/ðromos/<br>road	-	χωραφόδρομος<br>/xorafoðromos/	χωράφι<br>/chorafi/<br>field	δρόμος<br>/ðromos/<br>road
UN	PS	-	ουζοπαίδι<br>/ouzopeði/	ούζο<br>/ouzo/<br>Greek drink	παιδί<br>/peði/<br>child	-	φεγγαροπαίδι<br>/feggaropeði/	φεγγάρι<br>/fegari/<br>moon	παιδί<br>/peði/<br>child
UN	PS	-	αυγούπολη<br>/avɤopouli/	αυγό<br>/avɤo/<br>egg	πόλη<br>/poli/<br>city	-	αυγόφουσκα<br>/avɤofouska/	αυγό<br>/avɤo/<br>egg	φούσκα<br>/fouska/<br>balloon
UN	PS	-	φρουτόπορτα<br>/fteroporta/	φρούτο<br>/frouto/<br>fruit	πόρτα<br>/porta/<br>door	-	μπανανόπορτα<br>/bananoporta/	μπανάνα<br>/banana/<br>banana	πόρτα<br>/porta/<br>door
UN	PS	-	χρυσοπούλι<br>/chrisopouli/	χρυσό<br>/chriso/<br>gold	πουλί<br>/pouli/<br>bird	-	νεραϊδοπούλι<br>/neraiðopouli/	νεράιδα<br>/neraiða/<br>fairy	πουλί<br>/pouli/<br>bird

Note 1: T for Trained items, UN for Untrained items, TR for Transparent items, OP for Opaque items, PS for Pseudowords. Please, note that there is no meaning for PS.

Note 2: The frequencies are based on The Hellenic National Corpus (Institute of Language and Speech Processing, 2000) containing about 34,000,000 Greek words.

Appendix III

Table 1. Meaning compounds (pre- & post-test of the Intervention study) (N=16 items)

Trained items

	**Targets**	**Transcription**	**Translation**	**frequency**
T	πιατοθήκη	*piatothiki*	*dish-rack*	0
T	φρουτοσαλάτα	*froutosalata*	*fruit-salad*	3
T	θεατρόφιλος	*theatrofilos*	*theater-lover*	3
T	φυτοφάρμακο	*fitofarmako*	*pesticide*	12
T	ζωοτροφή	*zootrofi*	*food for animals*	13
T	καγκελόπορτα	*kagkeloporta*	*raildoor*	25
T	μοναχοπαίδι	*monachopeΔi*	*the only child*	26
T	ποδηλατόδρομος	*poΔilatoΔromos*	*bicycle-road*	0
T	χιονοπόλεμος	*chionopolemos*	*snow-war*	1
T	υπνοδωμάτιο	*ipnoΔomatio*	*bedroom*	32
T	σγουρομάλλης	*sγouromalis*	*with curly hair*	1
T	αναποδογυρίζω	*anapoΔoγirizo*	*turn upside-down*	1
T	θαλασσοπούλι	*thalossopouli*	*sea-bird*	6
T	μολυβοθήκη	*molivothiki*	*pencil case*	0
T	κουρτινόξυλο	*kourtinoxilo*	*curtain-wood*	1
T	μαρουλόφυλλο	*maroulofilo*	*lettuce- lead*	2

Note: The frequencies are based on The Hellenic National Corpus (Institute of Language and Speech Processing, 2000) containing about 34,000,000 Greek words.

Appendix IV

Table 1. Examples of pupils' responses in the Meaning categories

Meaning Categories	Definition of categories	Children's responses
Etymology (+)	Accurate answer:<br><br>The child reports both constituents of the compound.	Πιατοθήκη είναι η θήκη για τα πιάτα.<br>/Piatothiki einai i thiki ɣia ta piata/<br>A dish-rack is a rack for the dishes.
Semantics (+)	Accurate answer:<br><br>The child does not report the constituents of the compound.	Χιονοπόλεμο παίζουμε το χειμώνα.<br>/Chionopolemo pezoume to chimona/<br>We throw snowballs in winter.
Etymology (-)	Wrong answer:<br><br>The child reports both constituents of the compound.	Φυτοφάρμακο είναι ένα φάρμακο με φυτά.<br>/Fitofarmako ine ena farmako me fita./<br>Pesticide is a chemical made of plants (instead of saying:<br>Pesticide is a chemical for plants)
Semantics (-)	Wrong answer:<br><br>The child does not report constituents of the compound.	Κτηνοτρόφος είναι ένα πουλάκι.<br>/Ktinotrofos ine ena poulaki/<br>A stockbreeder is a small bird.
